# Principles of confounder selection

**DOI:** 10.1007/s10654-019-00494-6

**Published:** 2019-03-06

**Authors:** Tyler J. VanderWeele

**Affiliations:** Department of Epidemiology, Harvard T.H. Chan School of Public Health, 677 Huntington Avenue, Boston, MA 02115 USA

**Keywords:** Confounder, Causal inference, Collider, Covariate adjustment, Selection

## Abstract

Selecting an appropriate set of confounders for which to control is critical for reliable causal inference. Recent theoretical and methodological developments have helped clarify a number of principles of confounder selection. When complete knowledge of a causal diagram relating all covariates to each other is available, graphical rules can be used to make decisions about covariate control. Unfortunately, such complete knowledge is often unavailable. This paper puts forward a practical approach to confounder selection decisions when the somewhat less stringent assumption is made that knowledge is available for each covariate whether it is a cause of the exposure, and whether it is a cause of the outcome. Based on recent theoretically justified developments in the causal inference literature, the following proposal is made for covariate control decisions: control for each covariate that is a cause of the exposure, or of the outcome, or of both; exclude from this set any variable known to be an instrumental variable; and include as a covariate any proxy for an unmeasured variable that is a common cause of both the exposure and the outcome. Various principles of confounder selection are then further related to statistical covariate selection methods.

## Introduction

Confounding is a concern in almost all observational studies in epidemiology that focus on causality. Epidemiologic analyses are often criticized on the grounds that some third factor might be responsible for the relationship between the exposure and the outcome under study i.e., that the groups receiving and not receiving the exposure are different from one another in some other important variable that is also related to the outcome. As a result, considerable effort is often devoted during study design to consider what such confounding variables might be and to collect data on them. In the analysis of data, effort is made to control or adjust for such confounding variables. The hope is that by such efforts at data collection and analytic control, the groups with and without the exposure are in fact comparable within strata of such covariates. A critical question that arises in this context is how to go about deciding which covariates to select for control for confounding.

A formal system based on causal diagrams was put forward by Pearl [1, 2], which, if adequate knowledge with regard to the relevant underlying causal relationships is available, would suffice to make decisions with regard to confounding control [3, 4]. Unfortunately, in settings with numerous covariates, knowledge of a complete causal diagram, including the causal relationships amongst all the possible covariates themselves is often unavailable. Principles that are sometimes put forward for making these decisions when knowledge of a causal diagram is unavailable include, for example, (i) control for all pre-exposure measured variables or (ii) control for all common causes of the exposure and the outcome. While these principles are often helpful, it has been noted that in certain settings they can lead to controlling for a covariate that in fact introduces bias [4–9] or to not controlling for a covariate that would eliminate bias [9]. Decisions about confounding control are sometimes alternatively made solely on statistical grounds, for example, by examining whether controlling for a covariate changes an estimate by more than 10%, or by forward and backward selection, or by more contemporary machine learning methods. However, statistical analyses alone are not adequate for making decisions about confounder selection insofar as statistics alone generally cannot make determinations about temporal order. Statistical analyses cannot in general distinguish between confounders, which ought to be controlled for in the estimation of the total effect, versus mediators, which ought not be controlled for in the estimation of the total effect [10]. Some substantive knowledge is needed. Thus even for statistical variable selection techniques, the researcher must still make decisions as to what variables might at least potentially be considered a confounder (and e.g., not a mediator) before employing the statistical approaches.

This paper will put forward a synthesis of various relatively recent developments in causal inference surrounding the topic of confounder selection [1–18]. A criterion for determining what set of covariates to control for as confounders will be proposed, and various common statistical variable selection approaches will be discussed with regard to their adequacy in appropriately making confounding control decisions. The proposal in this paper is not intended to be definitive, but rather as (i) a way to attempt to make sense of the various developments concerning bias and confounding in causal inference, (ii) as a potentially practical and usable approach to confounder selection decisions, and (iii) as a starting point to generate further discussion, and potentially future refinements. We will first introduce some basic notation, then consider principles of confounder selection, and finally relate these to statistical covariate selection methods.

## Notation and definitions

Consider an exposure A and outcome Y, and measured covariates C. Let Y_a_ denote the counterfactual outcome or potential outcome that would have been observed for an individual if the exposure A had, possibly contrary to fact, been set to level a. We say that the covariates C suffice to control for confounding if the counterfactuals Y_a_ are independent of A conditional on C, which we denote by notation Y_a_ ⊥ A |C. The definition essentially states that within strata of C, the group that actually had exposure status A = a is representative of what would have occurred had the entire population with C = c been given exposure A = a. If this holds, we could use the observed data to reason about the effect of intervening to set A = a for the entire population.

This condition of no confounding for the effect of A on Y conditional on C is sometimes, in other literatures, referred to using different terminology. It is sometimes in epidemiology also referred to as “exchangeability” [19] or as “no unmeasured confounding” [20]; in the statistics literature it is sometimes referred to as “weak ignorability” or “ignorable treatment assignment” [21]; in the social sciences it is sometimes referred to as “selection on observables” [22, 23], or as “exogeneity” [23]. When this assumption holds and when we also have the technical consistency assumption that for those with A = a, we have that Y_a_ = Y, then we can estimate causal effects [2, 24], defined as a contrast of counterfactual outcomes, using the observed data and associations. Specifically we then have that:

$${\text{E}}\left[ {{\text{Y}}_{1} - {\text{Y}}_{0} \left| {{\text{c}}\left] { = {\text{E}}} \right[{\text{Y}}} \right|{\text{A}} = 1,{\text{c}}} \right] - {\text{E}}\left[ {{\text{Y}}|{\text{A}} = 0,{\text{c}}} \right]$$

**Fig. 1:**
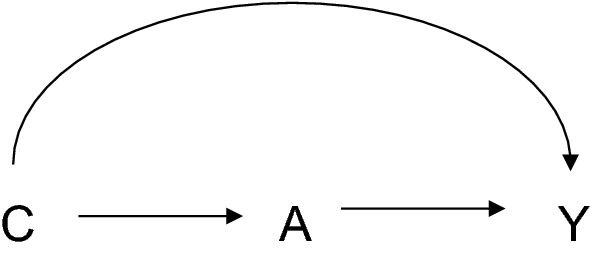
Confounding by covariates C of the relationship between exposure A and outcome Y

The left hand side of the equation is the causal effect of the exposure on the outcome conditional on the covariates C = c. The right hand side of the equation consists of the observed associations between the exposure and the outcome in the actual observed data. If the effect of A on Y is unconfounded conditional on the measured covariates C, as in Fig. 1, we can estimate causal effects from the observed data. The expression above is for causal effects on a difference scale, but if the effect of the exposure on the outcome is unconfounded conditional on covariates then one can likewise estimate the causal effect on the ratio scale from the observed data:

$${\text{P}}\left( {{\text{Y}}_{1} = 1|{\text{c}}} \right)/{\text{P}}\left( {{\text{Y}}_{0} = 1|{\text{c}}} \right) = {\text{P}}\left( {{\text{Y}} = 1|{\text{A}} = 1,{\text{c}}} \right)/{\text{P}}\left( {{\text{Y}} = 1|{\text{A}} = 0,{\text{c}}} \right)$$

We now turn to principles of confounder selection.

## Principles of confounder selection

The assumption of the absence of confounding is a strong one. With observational data we can never be certain that it holds. We attempt to control for covariates that are related to both the exposure and the outcome in order to make the assumption plausible. Causal diagrams can sometimes be helpful in this regard if something is known about the causal structure relating all of the variables to each other [2]. However, we must often make these decisions without having much knowledge of the underlying causal structures and without knowing for certain whether adjustment for a particular covariate will reduce bias. Different principles for deciding what covariates to adjust for to try to control for confounding may require different levels of knowledge regarding the nature of the covariates. If we truly had full knowledge of the structure of a causal diagram that related all of the covariates to each other and to the exposure and outcome then we could make use of the so-called “backdoor path criterion” of Pearl [1, 2] to determine which covariates would be sufficient to control for confounding bias. Without such detailed structural information about all of the different possible covariates, other approaches must then be used.

One principle of covariate selection for confounding control that is sometimes used is what might be referred to as the “pretreatment criterion” [25, 26]. In this approach one attempts to control for any variable that is prior to the treatment or exposure under study. Restriction is made to covariates that precede the exposure because otherwise such a covariate might be on the pathway from exposure to outcome and controlling for it might block some of the effect [10, 27].^1^ Any common cause of both the exposure and the outcome must be prior to the exposure and thus such restriction to pre-exposure covariates seems reasonable. Because we often do not know whether a particular covariate in fact affects both the exposure and the outcome, it may then seem best, whenever possible, to adjust for all available covariates that are prior to the exposure and indeed this approach has been advocated [25, 26] and is used with some frequency.

**Fig. 2:**
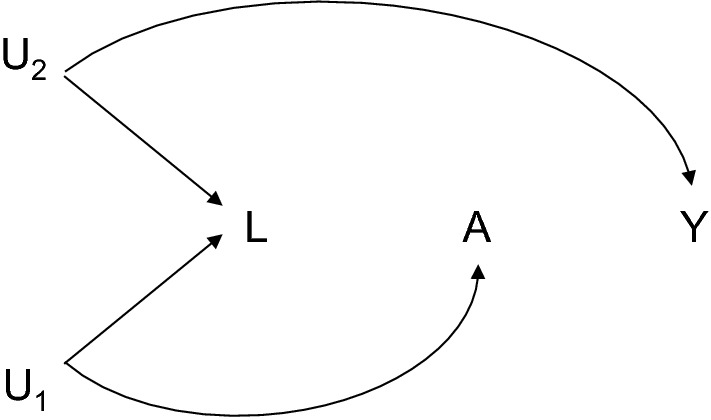
Controlling for pre-exposure covariate L introduces bias in the relationship between exposure A and outcome Y because L is a collider on the path from A to Y, since it is a common effect of U_1_ and U_2_

But is this “pre-treatment” approach to confounder selection the best? One problem that arises with the “pre-treatment” approach is that in principle one may end up controlling for a pre-exposure covariate that in fact introduces bias [2, 5–9]. In the causal diagram in Fig. 2, for example, an analysis of the association between A and Y without controlling for any covariates would give valid estimates of causal effects, but in an analysis adjusted for L, there would be bias because of the unblocked backdoor path A–U_1_–L–U_2_–Y that was unblocked by conditioning on the variable L [2, 7, 8]. In the causal diagram literature, the variable L that is a common effect of two variables on the path A–U_1_–L–U_2_–Y is sometimes referred to as “collider” and the bias induced by conditioning on the collider is sometimes referred to as “collider bias” or “M-bias” [2, 5–9]. In this setting, the “pre-treatment” confounder selection approach fails. Its use in fact introduces bias [2, 7].

**Fig. 3:**
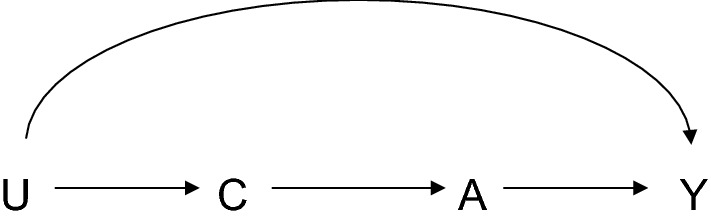
Controlling for measured covariate C, even in the presence of unmeasured variable U, eliminates confounding of the relationship between exposure A and outcome Y, even though C itself is not a common cause of A and Y

An alternative approach to confounder selection which requires relatively minimal knowledge of the underlying causal structure and is perhaps used with some frequency in practice in epidemiology is what one might call a “common cause” approach: one adjusts for all pre-exposure covariates that are common causes of exposure and outcome [28]. The application of this criterion requires somewhat more knowledge than the application of the “pre-treatment” criterion because one must have knowledge for each covariate whether it is a cause of the exposure and of the outcome, but still this required knowledge is considerably less than that required to employ the back-door path criterion which requires complete knowledge of the causal relations between each covariate and every other covariate. The common cause criterion has the advantage that if one is genuinely able to control for all common causes of the exposure and the outcome, then regardless of what the underlying causal diagram might be, control for this set of common causes will suffice to control for confounding for the effect of the exposure on the outcome [2]. The downside of the common cause criterion is that in certain instances, if data on some of the covariates that are common causes of the exposure and the outcome are not available, there might be a different set of covariates that suffices to control for confounding, but that is not captured by the common cause criterion. Consider, for example, the causal diagram in Fig. 3 and suppose that data on U is not available but that data on C is available. If the only covariate available were C, then, since C is not a common cause of A and Y, the common cause criterion would suggest not to control for it. However, if one did control for C, even though it is not a common cause of A and Y, this would suffice to control for confounding. So whereas the “pre-exposure” criterion was too liberal and could result in control for covariates that create bias, the “common cause” criterion is too conservative and may result in not controlling for covariates that in fact would suffice to eliminate bias.

An alternative approach that in some ways strikes an intermediate balance between these two alternatives is to control for any pre-exposure covariate that is a cause of the exposure, or the outcome, or both. We will refer to this criterion as the “disjunctive cause criterion” [9] because one controls for covariates that are causes of the exposure *or* the outcome (or are causes of both). Like the common cause criterion, this disjunctive cause criterion requires knowledge of whether each covariate is a cause of the exposure and whether it is a cause of the outcome, but it does not require knowledge of the full underlying causal diagram relating each of the covariates to all of the other covariates. The disjunctive cause criterion also has some attractive properties with regard to confounding control. The application of this criterion to Fig. 2 would result in not controlling for L since L is not a cause of A or Y; the application of the criterion would thus avoid bias generated by controlling for L in Fig. 2. Moreover, in Fig. 3 in a situation where U is unavailable, the disjunctive cause criterion would result in controlling for covariate C since C is a cause of Y; and the control for covariate C would then suffice to control for confounding and avoid the bias arising from the common cause criterion that results from not controlling for C. In fact, it can be shown, that for every causal diagram, if there is any subset of the measured covariates that suffices to control for confounding, then the set selected by the disjunctive cause criterion will suffice as well [9]. This property does not hold for the “pre-treatment” as illustrated by Fig. 2 and does not hold for the “common cause” criterion as illustrated in Fig. 3.^2^

**Fig. 4:**
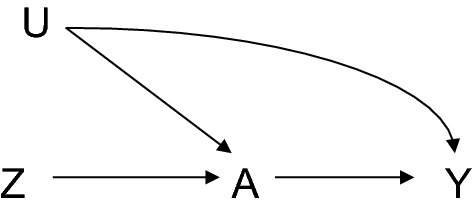
In the presence of uncontrolled confounding between exposure A and outcome Y induced by unmeasured variable U, controlling for the instrument Z can amplify the bias induced by U

A reasoned approach to confounding control, if knowledge is available on whether each covariate is a cause of the exposure and whether each covariate is a cause of the outcome, might then be to apply the disjunctive cause criterion and select those covariates that are causes of the exposure, or the outcome, or both. In light of the theoretical properties of this criterion it may be a sensible approach, but its use in practice would benefit from two further qualifications. First, it has been documented elsewhere that if there is some residual confounding due to an unmeasured covariate U, then controlling for a variable that is a cause of the exposure, but has no relation to the outcome except through the exposure, can in fact amplify the bias due to U [11–16]. For example, in Fig. 4, if U is unmeasured it will generate bias. However, in many cases, the bias will in fact be worse if adjustment is made for Z, than if adjustment is not made for Z [11–16]. Such a variable that is a cause of the exposure, but has no relation to the outcome except through the exposure is sometimes in other contexts called an “instrument” or an “instrumental variable” [29–31] and the additional bias that can result by controlling for an instrument in the presence of unmeasured confounding is sometimes called “Z-bias” [12, 16]. Instrumental variables can sometimes be useful in obtaining estimates of the causal effect through instrumental variable analysis [29–31], but controlling for instruments in a regression of the outcome on the exposure has the potential to generate additional bias. In general, it would thus be best in practice, if the disjunctive cause criterion is to be used, to discard any variable known to be an instrumental variable from covariate control. In general, the level of knowledge that is required to determine that a variable is an instrumental variable is considerable, as it must be known that it is a cause of the exposure but that it is otherwise completely unrelated to the outcome except through the exposure. It must be known then that the purported instrumental variable is not a direct cause of the outcome and that it is not related to the outcome through some other variable except through the exposure. Such substantive knowledge will often not be available, and when instruments are employed in instrumental variable analysis their use is often considered controversial. Thus, while it would be good to discard from covariate selection any covariate known to be an instrument, these settings might, in practice, be rare.

**Fig. 5:**
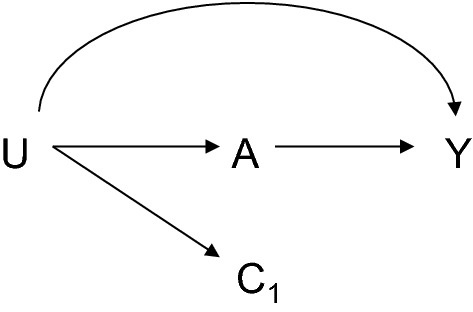
Control for a proxy confounder C_1_ of the true unmeasured confounder U will often, but not always, reduce confounding bias in the relationship between exposure A and outcome Y

A second qualification to the disjunctive cause criterion when used in practice is it might be desirable to adjust for any variable that does not satisfy the disjunctive cause criterion but that may be a proxy for a variable that does satisfy the criterion such as variable C_1_ in Fig. 5. A proxy for a variable that does satisfy the disjunctive cause may be essentially viewed as a confounder that is subject to measurement error, and in most cases adjustment for such variable will reduce the bias due to confounding [17, 18, 32, 33]. However, adjusting for a proxy of a confounder is not always guaranteed to reduce bias [18, 32, 33] and so care must be still taken and conclusions about effects are subject to somewhat more uncertainty. Methods for sensitivity analysis for unmeasured confounding can help assess how much residual confounding might be needed to explain away an effect estimate [34–39]. Cautions about controlling for “proxy confounders” are perhaps especially relevant in contexts in which the putative proxy confounder is in fact not a proxy for a common cause of the exposure and outcome, but rather a proxy for a cause of just the exposure, or of just the outcome, since if in fact it is a proxy in both of these senses then we are back to the confounding structure in Fig. 2 that can introduce collider bias. It thus may be best to restrict control for proxy confounders to those that are proxies for variables known to be a common cause of the exposure and the outcome.

Adding these two qualifications leads us to a summary principle for confounder selection of: control for each covariate that is a cause of the exposure, or of the outcome, or of both; exclude from this set any variable known to be an instrumental variable; and include as a covariate any proxy for an unmeasured variable that is a common cause of both the exposure and the outcome.

## Principles of confounder selection and confounder timing

Another consideration that should be taken into account when making decisions about confounder selection based on substantive knowledge is that of covariate timing. It was noted above that for estimation of total effects, rather than direct effects, we do not want to make adjustment for variables that may be on the pathway from the exposure to the outcome [2, 10, 27]. To avoid this, we often refrain from adjusting for covariates that occur temporally subsequent to the exposure. In many two-wave longitudinal studies, the exposure and covariates are all assessed at one time and the outcome is assessed at a subsequent time. However, in many cohort studies, data is collected on all exposures, covariates, and outcomes repeatedly across each wave, perhaps once per year, or once every 2 years. Such designs can allow researchers to examine the effects of time-varying exposures [40, 41], but even when assessing the effects of an exposure at a single point in time, such designs can help make more informed confounder selection decisions based on the temporal ordering of the data. One difficulty with studies in which the exposure and potential confounding covariates are all assessed at the same time is that it can be difficult to determine whether a covariate assessed at the same time as the exposure may in fact be affected by it.

Consider, for example, a study intended to assess the effect of physical activity on cardiovascular disease. Body mass index (BMI) might be available as a covariate and it may be thought important to then control for BMI as a confounder. However, it is of course also conceivable that BMI is on the pathway from physical activity to cardiovascular disease and that control for it may block some of the effect of physical activity. Conversely, it may also be the case that BMI itself affects both subsequent physical activity and subsequent incidence of cardiovascular disease. Someone with a very high BMI may have more difficulty regularly exercising. Thus it is possible that BMI is both a confounder (for the effect of subsequent physical activity) and also a mediator on the pathway from prior physical activity to cardiovascular disease. It is thus difficult to know whether or not to adjust for BMI if both BMI and physical activity are measured at the same time. We cannot adequately distinguish in this setting between confounding and mediation [10]. If, however, BMI is available repeatedly over time then it may be possible to control for BMI in the wave of data that is prior to the wave that uses exercise as the primary exposure. This would better rule out the possibility that the BMI variable used in the analysis is a mediator; if its measurement precedes that of physical activity by a year then it is more reasonable to interpret it as a confounder. When multiple waves of data are available it may thus be desirable to control for the covariates in the wave prior to the primary exposure of interest. It may also be desirable to control for prior levels of the exposure in the previous wave to further rule out confounding. This is not always an option when only two waves of data are available (one for the exposure and covariates and one for the outcome), but when multiple waves of data are available it can be possible to make decisions about covariate timing that allow one to control for confounders while better ruling out the possibility that one might in fact be controlling for a mediator. These considerations are certainly relevant in the context of the estimation of the causal effects of time-varying exposures but they are relevant even in the context of considering the effects of an exposure at a single point in time. It is also of course possible to carry out sensitivity analysis of the timing of confounder measurement, and to compare the results when confounders are controlled for contemporaneously with the exposures versus when they are controlled for in the prior wave [42–46].

## Statistical confounder selection

The approach described above for covariate selection can be useful when sufficient knowledge is available as to whether each covariate may be a cause of the exposure and/or the outcome. The approach described above essentially involves making decisions about confounder control based on substantive knowledge. Various data-driven statistical approaches to confounder selection have also been proposed. As will be discussed below, data-driven approaches do not obviate the need for substantive knowledge in confounder selection decisions, even though they are sometimes presented as stand-alone alternatives. Statistical data-driven approaches are sometimes motivated by the fact that there is far more covariate data that is available than is possible to adjust for in a standard regression model, especially when the number of covariates is relatively large and the sample size is relatively modest. Convergence properties of statistical models can then sometimes have very poor performance. A statistical covariate selection technique might then be useful in reducing the number of covariates to achieve a more parsimonious model. Traditionally, this was perhaps the primary motivation for statistical approaches to covariate selection. Alternatively, however, even when sample sizes are very large, if the number of covariates is also large it may be difficult to even go through each of the covariates one by one to assess whether they are causes of the exposure and/or outcome and this might also motivate a more statistically oriented approach to covariate selection. And, of course, both problems may be present: it may be impractical to substantively go through the covariates one-by-one to assess each and it may also be the case that the number of covariates may be large relative to, or even larger in absolute number than, the total sample size.

Historically, perhaps the most common statistical covariate selection techniques were forward and backward selection. In backward selection, one starts with the complete set of covariates and then iteratively discards each covariate unassociated with the outcome conditional on the exposure and the other covariates. It can be shown that if the total set of covariates suffice to control for confounding for the effect of the exposure on the outcome, and if backward selection at each stage does correctly select and discard covariates unassociated with the outcome conditional on exposure and all remaining covariates at that stage, then the final set of covariates selected will also suffice to control for confounding [9, 41]. In forward selection, one begins with an empty set of covariates and then examines associations of each covariate with the outcome conditional on the exposure adding the first covariate that is associated with the outcome, conditional on exposure; then at each stage one examines associations of each covariate with the outcome conditional on the exposure and the covariates already selected, adding the first additional covariate that is thus associated; the process continues until, with the set of covariates selected, all remaining covariates are independent of the outcome, conditional on the exposure and the covariates that had been selected. Again, provided the total set of covariates suffices to control for confounding for the effect of the exposure on the outcome, and that the forward selection at each stage does correctly identify the covariates that are and are not associated with the outcome conditional on exposure and all previously selected covariates at that stage, then under some further technical assumptions (that the distribution of the exposure, outcome, and covariates is “faithful” to the underlying causal diagram [2]), one can conclude that the final set of covariates selected will also suffice to control for confounding [9].

While the backward selection and forward selection procedures are intuitively appealing, they do suffer from a number of drawbacks when used in practice. First, when making the determination about whether a covariate is or is not associated with the outcome at each stage, statistical testing using p-values is often used in practice and such statistical testing of course in no way ensures that the correct conclusion is reached [47]. The confounding control properties above only hold if, at each stage the right decision is made. Second, once the final set of covariates is selected using either forward or backward selection, the most common approach is then to fit a final regression model with that set of covariates to obtain estimates and confidence intervals. Unfortunately, if the data have already been used to carry out covariate selection, the estimates and confidence intervals that are obtained following such selection are no longer valid [48]. The standard approaches to statistical inference, when used “post-selection”, break down. Recent work has examined approaches to carry out statistical inference after a data-based covariate selection procedure has been used, but these are no longer as straightforward as simply fitting a final regression model [49–51].

Alternatively, one might consider doing the covariate selection with half of the data and fitting the final model with the other half of the data but this results in considerable loss in the precision of the estimates, and standard errors are much larger, and confidence interval much wider, than they would otherwise be. A final disadvantage of backward selection when used in practice is that it requires that the sample size is sufficiently large to fit the initial model with all covariates included. If one is carrying out covariate selection because the initial set of covariates is very large, then it may not be possible to even begin with such backward selection approaches. Alternatively, if the sample size is sufficiently large that one can fit the initial model with all of the covariates then it might be sufficient to simply use that model to obtain estimates of the causal effect of the exposure on the outcome. Statistical covariate selection is then not even necessary. Because of these various reasons, these traditional approaches to covariate selection may be of somewhat limited value. With many covariates and a smaller dataset, forward selection might be used to try to determine a much smaller set of covariates for which to adjust in the final model, but, because of the post-selection statistical inference issues noted above, such analyses are perhaps best viewed as exploratory or hypothesis-generating, rather than as providing a reliable estimate of the causal effect.

A statistical approach to covariate selection closely related to forward and backward selection is what is sometimes called the “change-in-estimate” approach. In this approach covariate selection decisions are made based upon whether inclusion of a covariate changes the estimate of the causal effect for the exposure by more than some threshold, often 10% [48]. In some ways this is similar to the forward and backward selection approaches described above in examining empirical associations but uses the magnitude of the effect estimates (in particular the magnitude of the change in the exposure effect estimate) rather than the presence or absence of association, or threshold for a *p* value, in making covariate selection decisions. Like the forward and backward selection approaches based on associations or p-values, the change in estimate approach still requires that the initial total set of covariates suffice to control for confounding. If used independently one covariate at a time, without consideration of whether the set of covariates suffices to control for confounding, one may be led to control for a covariate that in fact generates bias, such as L in Fig. 2. Also, like the forward and backward selection approaches based on associations or p-values, validity of covariate selection with change in estimates requires that the decisions made about these association are correct, and that sampling variability does not lead to an incorrect decision about association. For example, one may end up with a change in the exposure coefficient with and without a covariate of more than 10%, not because the covariate is a confounder, but simply due to chance variation.

However, the change-in-estimate approach has one further disadvantage that the forward and backward selection procedures do not share: the change in estimate approach is relative to the effect measure and it is inappropriate for non-collapsible measures such as the odds ratio or hazard ratio if the outcome is common [52]. For non-collapsible measures such as the odds ratio or hazard ratio with a common outcome, marginal and conditional estimates are not directly comparable. Even in a randomized trial, one can have a true change in an odds ratio after controlling for a covariate, not because of confounding, but because of non-collapsibility [52]. Conversely, an odds ratio estimate may not change even after adjustment for a true confounder because for example, a downward change in the odds ratio effect measure induced by confounding may be balanced by an upward change in the measure due to non-collapsibility. Thus even beyond all of the caveats above concerning forward and backward selection, covariate selection based on change-in-estimate approaches is further problematic when non-collapsible effect measures are used.

An alternative approach to statistical covariate selection that has become popular is to use a procedure related to what is now sometimes called a “high-dimensional propensity score” [53, 54]. In this approach, one covariate at a time, one calculates the risk ratio between that covariate and the outcome, and for a binary covariate, one also examines the prevalence of the covariate comparing the exposed and unexposed. Using these quantities an approximate estimate of the bias that such a covariate might generate is obtained [53, 54] and covariates are prioritized in order of this approximate bias. Some portion of the covariates (e.g., 10%) are then chosen based on this ordering of the approximate bias. These might then also be supplemented with certain demographic covariates, or other covariates which, for various reasons, the investigator may want to force into the model. These covariates can then be used in covariate adjustment for the estimation of causal effects either through propensity scores [21, 53, 54], or through some other modeling approach. Compared to forward and backward selection, this approach has the advantage of in fact making use of information both on the magnitude of association each covariate has with the outcome and with the exposure, and effectively discarding those where one of these two is small. However, compared with the standard forward and backward selection procedures, it has the disadvantage of not sharing the theoretical property that the final resulting set of covariates is guaranteed to suffice to control for confounding if the initial total set suffices (provided the presence of associations is assessed accurately). The “high-dimensional propensity score” (HDPS) does not share this property with the traditional forward and backward selection approaches because with the HDPS, the selection is done one covariate at a time, independent of the others, rather that conditional on the others as with forward and backward selection. Its performance in practice may sometimes be reasonable, but its theoretical properties in no way guarantees this. Perhaps most importantly, however, the HDPS approach, like forward and backward selection, make no adjustment in statistical inference for the fact that the estimate in the final model are obtained “post-selection.”

Fortunately, more principled approaches to statistical covariate selection have begun to develop. Some of these involve the use of machine learning algorithms to carry out covariate selection and to carry out flexible modeling between the outcome, exposure, and covariates, and use cross-validation and other approaches to handle inference post-selection. An approach to covariate selection that is flexible and that has been used with some frequency in the biomedical sciences is targeted maximum likelihood estimation [55–57] which uses machine learning algorithms to model both the exposure and the outcome and cross-validation techniques to choose among the best models and covariates. While such approaches may hold tremendous promise for statistical covariate selection, more work is needed to understand the sample sizes and covariate numbers at which the approach is feasible and has reasonable small-sample properties. While the theoretical properties of these techniques are desirable, they are only necessarily applicable asymptotically (i.e., requiring large sample sizes to be guaranteed to hold), and their performance in smaller samples is sometimes less clear. More practical and simulation-based work on determining in what contexts such approaches to statistical covariate selection are feasible is needed. Moreover, even with the most sophisticated statistical covariate selection approaches, it still must be the case that the initial covariate set itself suffices to control for confounding, which of course requires some substantive knowledge involving the considerations discussed in the previous sections.

## Conclusion

I would thus propose that a practical and theoretically-informed approach to covariate selection would involve using the “disjunctive cause criterion” and thus choosing as confounders those variables that are causes of the exposure or outcome or both, then, additionally, discarding any variable known to be an instrumental variable, and including variables that do not satisfy the criterion but are good proxies for unmeasured common causes of the exposure and the outcome. This modified approach might be referred to as a “modified disjunctive cause criterion” and its use could then be accompanied by, depending on available sample size and number of covariates, either the use of a regression model controlling for all covariates chosen by the modified disjunctive cause criteria, or alternatively and perhaps preferably, when possible, the use of targeted maximum likelihood estimation [55–57] or other principled inferential machine learning approaches to choose both the relevant covariates, and the best flexible model fit. It is hoped that this proposal will be of some use in practice in obtaining more reliable estimates of causal effects, and will be the basis for further discussion and refinement.

## Footnotes

1 In principle one could control for covariates temporally subsequent to the exposure but not affected by the exposure [2], or even variables affected by the exposure but not related to the outcome [4] but since it is difficult to know for sure whether a covariate that is temporally subsequent to the exposure is affected by it, often the restriction is made to covariates prior to the treatment or exposure under study. It is possible that the variable occurs prior to the exposure but is measured retrospectively subsequent to the exposure, and such variables might also be considered, though concerns about measurement error of such variables might then also be introduced.

2 Another criterion that might be put forward that we could refer to as an “extended common cause criterion” would be to control for any variable that is either a common cause of the exposure and outcome, or that was on the pathway from such a common cause to the exposure or outcome. This criterion, like the disjunctive cause criterion, would select a sufficient set of confounders in both Figs. 2 and 3. The downside of this “extended common cause criterion” is that it requires far more knowledge of the underlying diagram. The “disjunctive cause criterion” and the “common cause criterion” only required knowledge of whether each variable is a cause of the exposure or of the outcome or of both. The “extended common cause criterion” requires also knowledge of whether each variable is such that there is another variable that is a common cause of the exposure and the outcome and for which the variable in question is on the pathway from the common cause to either the exposure or the outcome. In other words, the “extended common cause criterion” requires considerable knowledge of the relationships that potential covariates have to each other. It is difficult to conceive of contexts in which this information would be available without also having knowledge of the entire causal diagram; and with knowledge of the entire causal diagram, Pearl’s original backdoor path criterion would suffice.
